# Evaluation of Mood Disorder Questionnaire (MDQ) in Patients with Mood Disorders: A Multicenter Trial across China

**DOI:** 10.1371/journal.pone.0091895

**Published:** 2014-04-04

**Authors:** Hai-Chen Yang, Tie-Bang Liu, Han Rong, Jian-Qiang Bi, Er-Ni Ji, Hong-Jun Peng, Xiao-Ping Wang, Yi-Ru Fang, Cheng-Mei Yuan, Tian-Mei Si, Zheng Lu, Jian Hu, Zhi-Yu Chen, Yi Huang, Jing Sun, Hui-Chun Li, Chen Hu, Jin-Bei Zhang, Ling-Jiang Li

**Affiliations:** 1 Division of Mood Disorders, Shenzhen Mental Health Centre, Shenzhen Key Lab for Psychological Healthcare, Guangdong, China; 2 Mental Health Institute, The Second Xiangya Hospital, Central South University, Hunan, China; 3 Division of Mood Disorders, Shanghai Mental Health Center, Shanghai Jiaotong University School of Medicine, Shanghai, China; 4 Peking University Institute of Mental Health, Beijing, China; 5 Shanghai Tongji Hospital, Tongji University Medical School, Shanghai, China; 6 The First Hospital of Harbin Medical University, Heilongjiang, China; 7 Hangzhou Seventh People's Hospital, Zhejiang, China; 8 West China Hospital, Sichuan University, Sichuan, China; 9 The Affiliated Brain Hospital, Nanjing Medical University, Jiangsu, China; 10 The Second Affiliated Hospital, College of Medicine, Zhejiang University, Zhejiang, China; 11 Mood Disorders Center, Beijing Anding Hospital, Capital Medical University, Beijing, China; 12 The Third Affiliated Hospital of Sun Yat-Sen University, Guangdong, China; University of Adelaide, Australia

## Abstract

**Background:**

The aim of this study was to test the ability of the Chinese version of the Mood Disorder Questionnaire (MDQ) to identify Bipolar Disorders (BD) in patients diagnosed with Major Depressive Disorder (MDD) or Unipolar Disorder (UD) in the clinical setting.

**Methods:**

1,487 being treated for MDD or UD at 12 mental health centers across China, completed the MDQ and subsequently examined by the Mini International Neuropsychiatric Interview (MINI). Receiver Operating Characteristic(ROC) curves were used to determine the ability of the MDQ to differentiate between BD (BD, BD-I and BD-II) and MDD or UD and patients with BD-I from patients with BD-II.

**Results:**

Of the 1,487 patients, 309 (20.8%) satisfied the DSM-IV criteria for BD: 118 (7.9%) for BD-I and 191 (12.8%) for BD-II. When only part one of the MDQ was used, the best cutoff was 7 between BD and UD (sensitivity 0.66, specificity 0.88, positive predictive value 0.59, negative predictive value 0.91), 6 between BD-II and UD, and 10 between BD-I and BD-II. If all three parts of the MDQ were used, the MDQ could not distinguish between BD and UD at a cutoff of 7 (or 6), and the sensitivity was only 0.22 (or 0.24).

**Conclusion:**

The Chinese version of the MDQ had good psychometric features in screening bipolar disorders from depressive patients with mood disorders when part two and part three of the MDQ were ignored.

## Introduction

Bipolar disorder (BD), also known as bipolar affective disorder or manic-depressive disorder. Individuals with BD experience episodes of an elevated or agitated mood known as mania/hypomania alternating with episodes of depression. Bipolar disorders have two main subtypes, bipolar I disorder (BD-I), bipolar II disorder (BD-II).The diagnosis of BD-I need one or more manic episodes. A depressive episode is not required for BD-I diagnosis, but it frequently occurs. The diagnosis of BD-II need one or more hypomanic episodes (without manic episode) and one or more major depressive episode. Bipolar disorder not otherwise specified (BD-NOS) is a catchall category, diagnosed when the disorder does not fall within a specific subtype. Hypomanic episode do not go to the full extremes of mania (*i.e.*, do not usually cause severe social or occupational impairment, and are without psychosis), and this can make BD-II more difficult to diagnose, since the hypomanic episodes may simply appear as a period of high productivity and creativity.

BD is a common disorder. Lifetime prevalence estimates are 1.0% for BD-I, 1.1% for BD-II, and 2.4% for subthreshold BD in the general adult population (aged ≥18 years) in the United States [1]. In a large community cohort in Europe, the prevalence of bipolar disorder (BD-I and BD-II) based on the DSM-IV criteria was 5.5% [2]. Bipolar disorders cause role impairment and high mortality and economic burden [3]. In practice, many patients with BD experience a delay in initiating treatment or are given the wrong treatment because of the under-recognition and frequent misdiagnosis of this disorder [4]–[6]. Of the self-rating mania/hypomania questionnaires developed to screen for BD [7], [8], the Mood Disorder Questionnaire (MDQ) is one that has been used effectively in many countries [7], [9]–[14].

The MDQ consists of three parts. In part one, the MDQ screens for a lifetime history of manic/hypomanic symptoms using 13 yes/no items. The second part asks whether two or more manic/hypomanic symptoms have been experienced during the same period. Part three assesses the level of functional impairment due to the symptoms on a 4-point scale (“no” to “severe”) [7], [15]. There are disagreements with respect to the scoring of the MDQ. Hirschfeld et al (2000) recommended that the positive response to MDQ required the presence of at least seven symptoms that co-occur and caused moderate to severe impairment due to the manic/hypomanic symptoms [7]. Some studies [9], [11] have found that the MDQ may be insensitive in the detection of BD due to the items in part three. Some studies have proposed modifying the MDQ scoring by ignoring part three and lowering the threshold screening for BD. The patients' assessments of functional impairment in the part three were influenced mostly by their insight, an area that is typically impaired in the patients with BD [16]–[18]. Secondly, because impairment in functioning is not necessary to diagnose hypomania, requiring impairment on the MDQ to determine BD will reduce its sensitivity for detecting BD-II [19]. In an earlier and smaller-sample Chinese study, in which the threshold number of symptom items co-occurring in the same time period and causing moderate or severe impairment was not rated, patients with a total score equal to or higher than 7 were identified as potentially suffering from BD [13]. It was not clear if all three parts of the MDQ could be used in clinical settings in China.

In most studies concerning the MDQ, including the earlier study in China, testing of subjects with mood disorders was not restricted to the depressive phase [7], [9]–[13]. Still, mood phases may have an impact on the results of these self-rating questionnaires [19], [20]. Patients with BD in the depressive phase have difficulty recalling past manic/hypomanic symptoms [21]. In addition, a depressive episode is usually the first mood syndrome at the onset of BD, and depressive episodes are more frequent than manic/hypomanic episodes [22], often leading to the misdiagnosis of BD as major depressive disorder (MDD), also called unipolar depression (UD). Therefore, it was necessary to study the response of the patients with mood disorders to the MDQ during their depressive phase.

We conducted this study to determine (1) which parts of the Chinese version of the MDQ should be used to screen for BD in the clinical setting; (2) compare the use of the MDQ for patients during the depressive phase with a previous study in which the MDQ was administered during any phase (mania, depression) and (3) determine if the results of our previous study of the MDQ in two psychiatric hospitals could be replicated in other centres which included psychiatric clinics and general hospitals across China.

## Methods

### Study participants and settings

The Diagnostic Assessment Service for People with Bipolar Disorders in China (DASP) is an ongoing national study initiated by the Chinese Society of Psychiatry (CSP) with the aim of developing and testing the usefulness of screening tools for BD in patients treated for MDD. The first survey of the DASP project was carried out in 13 major psychiatric hospitals and units of general hospitals between September 1, 2010 and February 28, 2011. These settings were evenly distributed in China and served both catchment area patients and patients from neighboring areas. Both in- and outpatients experiencing a major depressive episode were enrolled if they were between 16 and 65 years of age, had a DSM-IV or ICD-10 diagnosis of MDD based on a review of their medical records, understood the aims of the study and provided informed consent. Exclusion criteria included a past diagnosis of BD, ongoing significant medical or neurological condition(s), depressive disorders secondary to a general medical or neurological condition, or having received electroconvulsive therapy (ECT) in the past month. The study protocol was approved by the Clinical Research Ethics Committees of the respective study centers. All patients who participated in this study completed written consent forms.

### Instrument and assessment procedure

In- or outpatients in a depressive state with a diagnosis of MDD who were receiving treatment in the participating hospitals/units were referred by their treating psychiatrists to the member of the research team based at their particular site and screened for eligibility. All members of the research team were qualified psychiatrists. Patients fulfilling the study criteria were invited to participate in the study.

The patients' basic socio-demographic data were collected with a questionnaire designed for the study in a clinical interview, supplemented by a review of their medical records (Table 1). The diagnostic assessment of BD was conducted with the validated Chinese version of the Mini International Neuropsychiatric Interview (MINI) (Version 5.0) to establish DSM-IV BD-I/BD-II diagnoses [23], [24].

**Table 1:** Description of samples.

		BD-I	BD-II	UD	*P*
Number		118	191	1178	
Age		35.43	35.46	40.54	<0.01
Sex	Male	51	97	385	<0.01
Marriage state	Married	76	116	824	= 0.01
	Unmarried	37	68	288	
	Divorced	5	7	47	
	Loss	0	0	19	
Job	Employed	68	110	617	>0.05
	Retired	8	26	195	
	Unemployed	42	55	366	
Education	Lower than senior high school	31	52	356	= 0.48
	Senior high school Equal to a	32	44	312	
	Bachelor's degree	47	85	465	
	Postgraduate	8	10	45	

The MINI is a short, structured diagnostic interview that was developed jointly by psychiatrists and clinicians for DSM-IV psychiatric disorders. It was designed to meet the need for a short (approximately 15 minutes) but accurate structured psychiatric interview for multicenter clinical trials [23]. The Chinese version of the MINI showed strong reliability and validity in eliciting symptom criteria used to make DSM diagnoses [24]. The Chinese version of the MDQ (C-MDQ) has been validated in China [13]. The internal consistency (Cronbach's alpha) of the C-MDQ was 0.79. Sensitivity was 0.64, specificity was 0.80 and the area under curve was 0.75 at the optimal screening cutoff between BD and UD [13].

All twelve raters in this study were trained in diagnosing BD using the MINI in 20 MDD patients prior to the study. In the reliability exercise, raters' judgments of BD were compared with the best estimate clinical diagnoses [25]; the kappa values were above 0.85 for each rater. Patients younger than 18 years were included as long as they verbally agreed to participate and written consent was obtained from patients or guardians. After giving consent, patients were invited to complete the C-MDQ. Patients then underwent a DSM-IV diagnostic interview using the MINI by a rater who was blind to the C-MDQ results.

### Statistical analysis

Data were analyzed using the SPSS package, Version 17.0. One-way analysis of variance (ANOVA) and t-tests were used to compare the MDQ scores among the mood disorder patients. The receiver operating characteristic (ROC) curve was used to determine whether the patients with different mood disorders (BD, BD-I, BD-II, MDD) could be differentiated and to ascertain the sensitivity (SEN) and specificity (SPE) at various cutoffs. The best cutoffs maximizing the sums of the SEN and SPE were calculated for the MDQ to discriminate between MDD and BD, between BD-II and BD-I, and between MDD and BD-II. The criterion validity of the MDQ was estimated using the SEN, SPE, positive predictive value (PPV), negative predictive value (NPV) and the area under the curve (AUC).

## Results

### 1. Description of samples

Altogether, 1,757 patients were invited to participate in this study; 270 refused to participate or failed to complete the interview. There were no significant differences between the enrolled patients and patients who did not participate in terms of age and sex. Ultimately, 1,487 patients were included in the analysis. Of the 1,487 patients, 309 (20.8%) satisfied the DSM-IV criteria for BD: 118 (7.9%) for BD-I and 191 (12.8%) for BD-II. Table 1 displays the socio-demographic characteristics of the whole sample and separately for patients by diagnosis.

### 2. MDQ scores

The mean MDQ score was 7.29 (SD 3.23) for BD, 8.01 (SD 3.44) for BD-I, 6.85 (SD 3.02) for BD-II and 2.91 (SD 2.75) for UD. There was a significant difference among the scores of depressive patients with BD-I, BD-II or UD based on one-way analysis of variance (*P*<0.01, ANOVA). The mean MDQ score for BD patients was significantly higher than that of UD; BD-I was higher than BD-II, and BD-II was higher than UD (t-test, *P*<0.01).

### 3. Only part one of MDQ used

#### 3.1. ROC curve analysis between BD and UD

**Figure 1:**
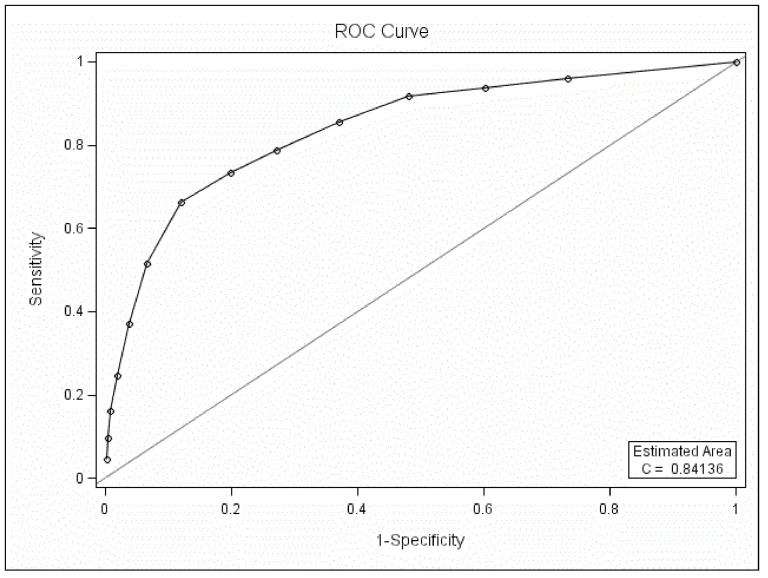
ROC curve analysis between BD and BD with only part one of the MDQ scored.

The MDQ could differentiate BD patients from UD patients when only part one was used in the ROC curve analysis (*P*<0.05, Figure 1). The AUC was 0.84. The best screening cutoff between BD and UD was 7 (SEN 0.66, SPE 0.88, PPV 0.59, NPV 0.91).

#### 3.2. ROC curve analysis between BD-I I and UD

**Figure 2:**
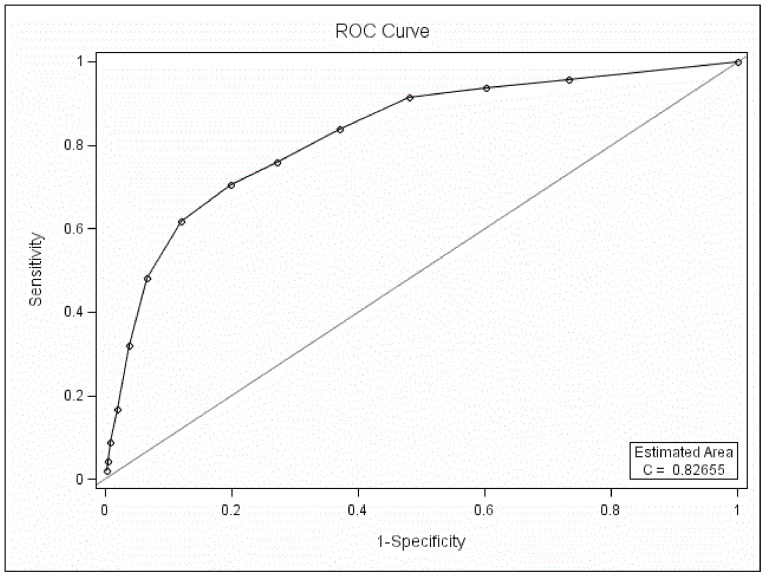
ROC curve analysis between BD-II and UD with only part one of the MDQ scored.

The MDQ could differentiate BD-II patients from UD patients when only part one was used in the ROC curve analysis (*P*<0.05, AUC 0.83, Figure 2). The best screening cutoff between BD-II and UD was 6 (SEN 0.71, SPE 0.80, PPV 0.37, NPV 0.94).

Because the mean MDQ score of the patients with BD-I was highest and that of UD was lowest, and MDQ could screen BD-II patients from UD patients by ROC analysis, we did not compare BD-I and UD in the ROC analysis.

#### 3.3. ROC curve analysis between BD-I and BD-II

**Figure 3:**
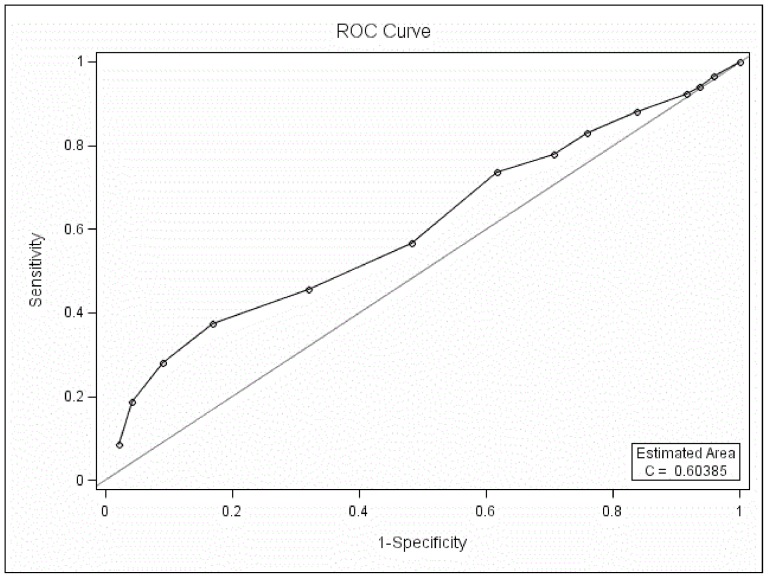
ROC curve analysis between BD-I and BD-II with only part one of the MDQ scored.

The MDQ could differentiate BD-I patients from BD-II when only part one was used in the ROC curve analysis (*P*<0.05, AUC 0.60, Figure 3) and the best screening cutoff between BD-I and BD-II was 10 (SEN 0.37, SPE 0.83, PPV 0.58, NPV 0.59).

Compared to the higher AUC between BD and UD (0.84), between BD-II and UD (0.83), the AUC between BD-I and BD-II is low (0.60) and might limit its usefulness.

### 4. All three parts of MDQ used

#### 4.1. If the cutoff was 7 between BD and UD

**Figure 4:**
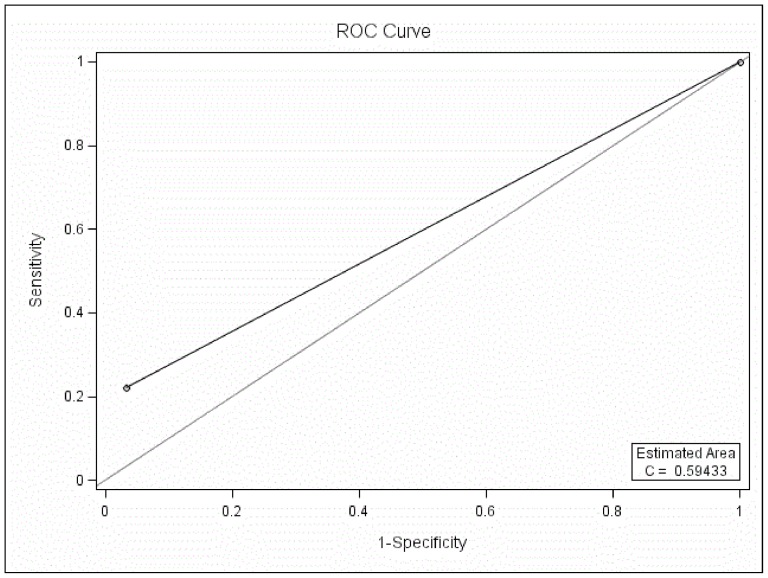
ROC curve analysis between BD and UD at the cutoff of 7 with three parts of the MDQ scored.

The MDQ could not differentiate BD patients from UD patients at the cutoff of 7 when all three parts of the MDQ were used in the ROC curve analysis (*P*>0.05, AUC 0.59, SEN 0.22, SPE 0.97, PPV 0.65, NPV 0.83, Figure 4).

#### 4.2. If the cutoff was 6 between BD and UD

**Figure 5:**
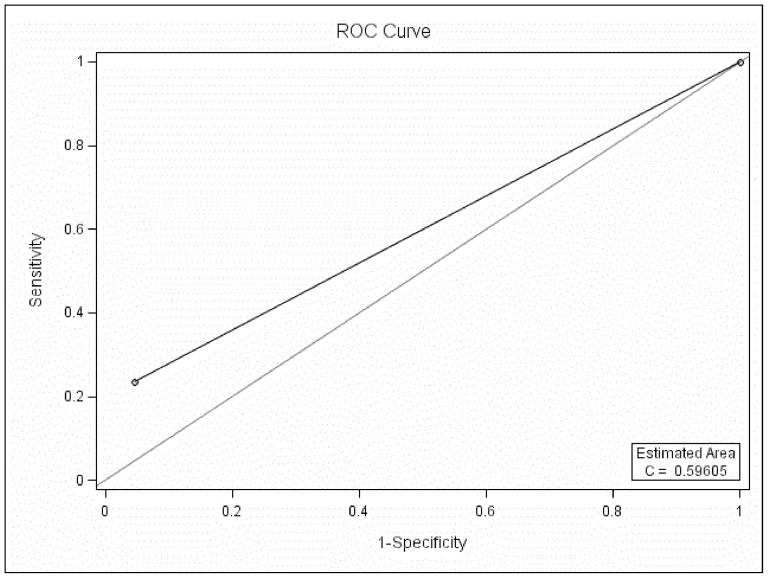
ROC curve analysis between BD and UD at the cutoff of 6 with three parts of the MDQ scored.

If the best cutoff of 6 between BD-II and UD was used as the optimal screening cutoff between BD and UD, the MDQ could not differentiate BD patients from UD when all three parts of the MDQ were used in the ROC curve analysis (*P*>0.05, AUC 0.60, SEN 0.24, SPE 0.96, PPV 0.58, NPV 0.83, Figure 5).

## Discussion

In this study, we found that if only part one of MDQ was used, MDQ could differentiate between BD and UD, between BD-II and UD, and between BD-I and BD-II. Because the treatments of different types of mood disorders and subtypes of bipolar disorders are different, MDQ which help clinicians in differentiating mood disorders is useful in practice.

The MDQ scores of depressive patients with BD-I were higher than those with BD-II, which were higher than for those with UD. The MDQ scores of depressive patients with mood disorders were lowest in patients with UD, followed by BD-II and BD-I. The change of MDQ score in patients with mood disorders was similar to that of the earlier Chinese study, which did not limit patients to the depressive phase [13].

When only part one of the MDQ was used, the best screening cutoff between depressed patients with BD and depressed patients with UD was 7 in this study. The cutoff was identical in most of the MDQ studies conducted worldwide, including the earlier study in China in which patients could be in any mood phase [7], [9], [12]–[14]. The feature of relatively low sensitivity (0.66) and higher specificity (0.88) in this study was similar to that of other studies as well [7], [9], [13]–[14]. The stable cutoff between BD and UD suggested that the impact of the type of mood episode for the patients with mood disorders on the MDQ score was minimal and could be ignored.

Again, when only part one of the MDQ was used, the best screening cutoff between BD-I and BD-II was 10, a different finding from the cutoff of 8 in the earlier study from China. The best screening cutoff between BD-II and UD was 6, while the cutoff was 5 in an earlier study [13]. Although the definite cutoffs were different, this study demonstrated again that the MDQ could screen patients with BD-I from patients with BD-II, and patients with BD-II from UD [13]. The low AUC (0.60) between BD-I and BD-II might limit its usefulness compared to the higher AUC (0.83) between BD-II and UD.

When all three parts were used, the MDQ could not be used as a screening tool in clinical settings. Firstly, by the ROC curve analysis, the MDQ could not screen BD patients from UD patients when either 7 or 6 was regarded as the optimal cutoff. We suggest that the best screening cutoff between BD-II and UD could also be used as the optimal cutoff between BD and UD to improve screening for BD-II [13]. Secondly, the sensitivities 0.22 (or 0.24) were too low to screen at cutoffs 7 (or 6) for a screening tool. Our results were similar to the results from a study in Korea. Kim et al (2008) found that a modified scoring of the MDQ (ignoring questions on the co-occurrence of symptoms and functional impairment) yielded an SEN of 0.68 and an SPE of 0.63 for BD, whereas the values were 0.29 and 0.77, respectively, using the standard MDQ scoring [9]. The results of the Korean study were similar to ours. Therefore, we suggested that only part one of the MDQ (13 items of manic/hypomanic symptoms) should be scored if the MDQ is to be used as a screening tool for the patients with mood disorders in clinical settings.

There were two limitations in the study. Firstly, we could not compare the severity of depression between patients because no standardized instrument was used to measure the severity of depressive symptoms. Secondly, depressed patients who had a previous diagnosis of BD were excluded from the study. The subjects with BD in this study may not be representative of all BD patients.

### Conclusions

Firstly, the C-MDQ, using only part one, is suitable to screen for BD in clinical settings. Secondly, the MDQ results for mood disorder patients in the depressive phase were similar to those of mood disorder patients in any mood phase. Thirdly, the results of this multicenter study were similar to those of the earlier study that was limited to two psychiatric hospitals in China.
